# Telomere Length in Human Spermatogenic Cells as a New Potential Predictor of Clinical Outcomes in ART Treatment with Intracytoplasmic Injection of Testicular Spermatozoa

**DOI:** 10.3390/ijms241310427

**Published:** 2023-06-21

**Authors:** Anna A. Pendina, Mikhail I. Krapivin, Yanina M. Sagurova, Irina D. Mekina, Evgeniia M. Komarova, Andrei V. Tikhonov, Arina V. Golubeva, Alexander M. Gzgzyan, Igor Yu. Kogan, Olga A. Efimova

**Affiliations:** D.O. Ott Research Institute of Obstetrics, Gynecology and Reproductology, Mendeleevskaya Line 3, 199034 Saint Petersburg, Russia

**Keywords:** telomeres, Q-FISH, spermatogonia, spermatocytes I, human spermatogenesis, azoospermia, TESE, ICSI

## Abstract

Predicting the clinical outcomes of intracytoplasmic sperm injection (ICSI) cycles that use the testicular spermatozoa of azoospermic patients presents a challenge. Thus, the development of additional approaches to assessing the competence of a testicular-sperm-derived embryo without causing damage to gametes or the embryo is necessary. One of the key parameters in determining such developmental competence is telomere length (TL). We aimed to analyze TLs in spermatogenic cells from the testicular biopsy samples of azoospermic patients and determine how this parameter influences embryo competence for pre- and post-implantation development. Using Q-FISH, we studied the TL of the chromosomes in spermatogonia and spermatocytes I from the TESE biopsy samples of 30 azoospermic patients. An increase in TL was detected during the differentiation from spermatogonia to spermatocytes I. The patients’ testicular spermatozoa were used in 37 ICSI cycles that resulted in 22 embryo transfers. Nine pregnancies resulted, of which, one was ectopic and eight ended in birth. The analysis of embryological outcomes revealed a dependence between embryo competence for development to the blastocyst stage and the TL in spermatogenic cells. The TLs in spermatogonia and spermatocytes I in the testicular biopsy samples were found to be higher in patients whose testicular sperm ICSI cycles resulted in a birth. Therefore, the length of telomeres in spermatogenic cells can be considered as a potential prognostic criterion in assessing the competence of testicular-sperm-derived embryos for pre- and post-implantation development. The results of this study provide the basis for the development of a laboratory test for the prediction of testicular sperm ICSI cycle outcomes.

## 1. Introduction

Despite the rapid development of assisted reproductive technologies (ART), their efficiency leaves room for improvement, especially when it comes to the use of testicular spermatozoa [1,2,3]. The success rate of fertilization with testicular spermatozoa fluctuates between 14.5% and 28% [4,5]. Unsuccessful ART outcomes are associated with disturbances in any of the stages, from fertilization failure to arrested embryo development in the pre- and post-implantation stages. Consequently, predicting ART outcomes is an extremely challenging but particularly meaningful task.

The low efficiency of testicular spermatozoa ART cycles determines the need for developing additional methods of assessing embryo developmental potential without damaging gametes or embryos. Telomere length (TL) is among the key parameters determining said potential [6,7,8]. Telomeres are ribonucleoprotein complexes at the ends of human chromosomes, consisting of a varying number of tandem repeats of hexanucleotides, telomeric RNA, and shelterin-complex proteins [9,10,11]. The primary function of telomeres is to protect the ends of chromosomes from merging, recognition by nucleases and non-homologous recombination [12,13]. Telomeres are among the most dynamic structural units of the human genome. On the one hand, their length decreases during ontogenesis. This process unavoidably occurs in the course of mitotic activity due to DNA end under-replication [6,8,14,15,16] and/or due to telomere damage caused by the detrimental impact of multiple external factors, mostly facilitated by a heightened content of reactive oxygen species [17,18,19]. On the other hand, TL increases during the transmission of hereditary information into the next generations, mostly in gametes and early embryos through the activity of telomerase and/or the mechanisms of the alternative lengthening of telomeres (ALT) [20,21,22]. Therefore, telomere length rests in a permanent dynamic balance. Maintaining this balance is crucial because its violation can result in critical telomere shortening and cell death.

In a newly formed organism, the significance of TL, which is determined by that of the sperm and the oocyte, is self-evident. It is the TL that determines a cell’s capacity for intensive mitotic activity, which is necessary for successful embryo development. Previously, we established that the length of telomeres in sperm-derived chromosomes in a human zygote significantly exceeds that in oocyte-derived chromosomes [23]. Consequently, several questions arise: At which stage of spermatogenesis do telomeres change in length? How do TLs across spermatogenic cells correlate with one another? Does telomere length in male spermatogenic cells impact pre- and post-implantation development? Not only has the adoption of testicular sperm extraction (TESE) in medical practice enabled the use of testicular spermatozoa for fertilization, but it has also opened up opportunities to investigate various characteristics of testicular tissue, including TLs in spermatogenic cells [24,25,26,27,28,29]. The present study aimed to investigate TLs in spermatogonia and spermatocytes I, as well as their impact on the competence of testicular-sperm-derived embryos for pre- and post-implantation development. The results of this study can form the basis for the development of a test for predicting ART outcomes in cases of fertilization using testicular spermatozoa.

## 2. Results

### 2.1. Patients and Samples

**Figure 1:**
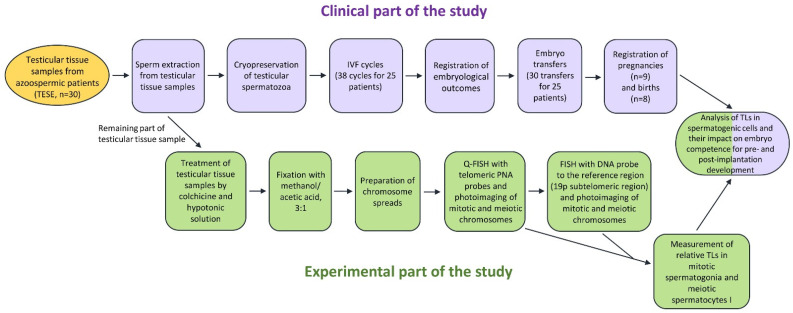
The flowchart of the study.

The paper investigated the TL in mitotic and meiotic chromosomes from spermatogonia and spermatocytes I obtained from testicular sperm extraction (TESE) biopsy samples collected from 30 azoospermic patients. The flowchart of the study is presented in Figure 1.

The TESE was conducted to obtain testicular spermatozoa for future use in the intracytoplasmic sperm injection (ICSI) procedure as part of the assisted reproductive technologies. Spermatozoa were detected in 29 of the testicular tissue samples, with their number in the field of view varying from one to ten and more. The testicular tissue sample of one of the patients did not contain detectable spermatozoa. The demographic details of the participants and their female partners, the number of spermatozoa in the biopsy sample field of view, the data on embryological outcomes, and the occurrence of pregnancy and birth are contained in Table 1.

### 2.2. TL Analysis in Mitotic Chromosomes of Spermatogonia and Meiotic Chromosomes of Spermatocytes I in Azoospermic Patients’ Testicular Tissue Samples

**Figure 2:**
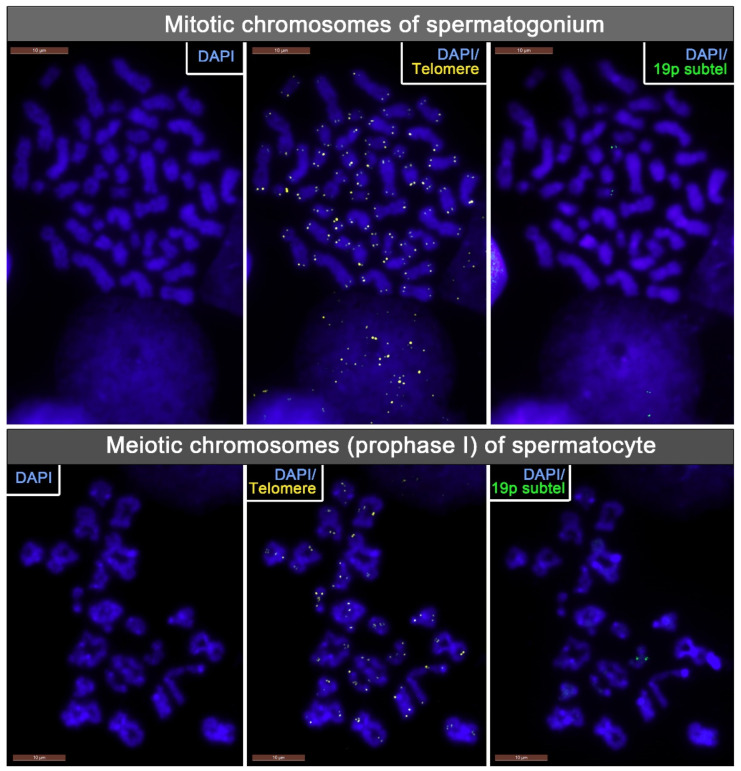
Mitotic metaphase chromosomes from human spermatogonium and meiotic prophase chromosomes from human spermatocyte from an azoospermic patient’s testicular tissue sample. Telomeres and 19p subtelomeres were detected through fluorescent in situ hybridization (FISH), and the chromosomes were stained with DAPI.

TLs were assessed on cytogenetic preparations of mitotic chromosomes from spermatogonia and meiotic chromosomes of spermatocytes I. We ensured reliable identification of the mitotic metaphase chromosomes from the spermatogonia and meiotic prophase chromosomes from the spermatocytes I by treating the testicular biopsy fragments with a hypotonic solution with the addition of colchicine (Figure 2). TLs were assessed through quantitative fluorescent in situ hybridization (Q-FISH) with PNA probes to telomeric sequences. Q-FISH is the optimal technique for studying the TLs of individual chromosomes, both mitotic and meiotic.

To mitigate the impact of chromosome condensation levels on the results, we calculated relative instead of absolute TLs. Relative TLs were measured as a ratio of the fluorescence intensity of the hybridization signal to telomeric regions to a reference hybridization signal. As a reference, we used the DNA probe to the subtelomeric region of the short arm of chromosome 19 (19p), which is characterized by an exceptionally low interindividual variability. To objectify the results, we assessed the TL of chromosome 19 across all of the analyzed spermatogonia and spermatocytes I after reliably identifying them with the reference probe. Therefore, we assessed the relative TL of the same chromosome across all of the analyzed cells.

To obtain a relative TL value for each mitotic spermatogonium, we measured telomeric fluorescence in each sister chromatid of the long and short arm of chromosome 19 (four signals) and calculated the average value. Then, we divided this value by the average value of 19p subtelomeric fluorescence calculated from two measurements made on sister chromatids of the same chromosome 19. The same measurements were performed for the second homologue of chromosome 19 to further find the average value of relative TL in each spermatogonial chromosome set. To obtain the relative TL value for each meiotic spermatocyte I, we used the same algorithm with the only difference being that telomeric and subtelomeric FISH signals were measured in chromosomes 19 bivalents. All further comparisons were drawn based on the assessed TLs of the long and short arms of chromosome 19.

To justify the possibility of this approach in our earlier studies [23,30], we characterised the strength of interrelation between the TL of an individual chromosome and that of other chromosomes on the same metaphase plate, based on correlation analysis. The analysis showed a strong correlation between the TL of an individual chromosome and that of other chromosomes on the metaphase plate: Spearman’s test, ρ = 0.8351, *p* < 0.0001 [23], and ρ = 0.915, *p* < 0.0001 [30], thus evidencing the applicability of this approach.

**Figure 3:**
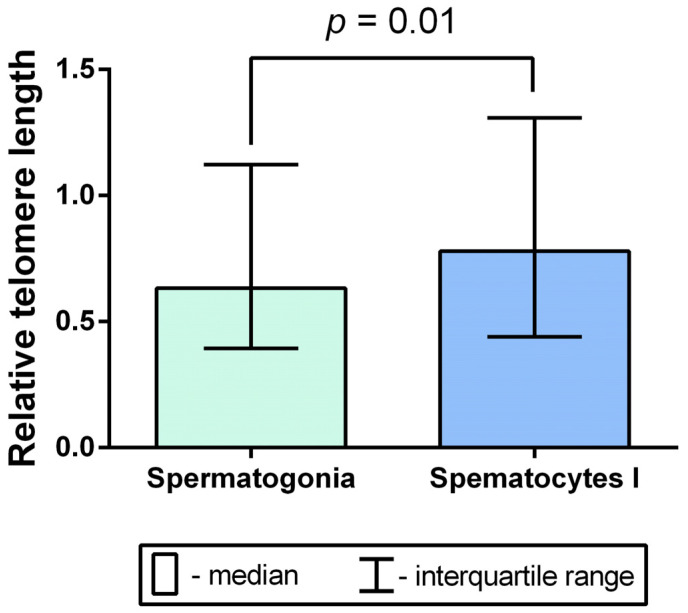
A comparison of mean relative telomere length of chromosomes in spermatogonia and spermatocytes I obtained from the testicular biopsy samples of 30 azoospermic patients (Mann–Whitney U test, *p* = 0.01).

We calculated relative TLs in 441 mitotic spermatogonia and 351 meiotic spermatocytes I. A total of 3528 telomeric and 1764 subtelomeric 19p FISH signals were measured in spermatogonia, and a total of 2808 telomeric and 1404 subtelomeric 19p FISH signals were measured in spermatocytes I. The comparative analysis of relative TLs of mitotic chromosomes in spermatogonia and meiotic chromosomes in spermatocytes I showed a significant increase in the latter (Mann–Whitney U test, *p* = 0.01) (Figure 3).

Therefore, in the course of spermatogenesis, TL increases during the transition from the spermatogonia stage to the spermatocyte stage.

### 2.3. A Comparison of Intraindividual and Interindividual Relative TL Variability in Spermatogonia and Spermatocytes I

To assess the intraindividual (intercellular) relative TL variability in spermatogonia and spermatocytes I, we first assessed relative TLs in the individual cells of each type in the 30 patients’ biopsy samples. We then calculated the mean value for all cells of one type in the sample, calculated the deviation from the obtained mean value for each cell, and expressed the obtained value as a number greater than 1 (that is, for values less than 1, we found the inverse of 1/x). 

To assess the interindividual TL variability, we determined the mean relative TL for both spermatogonia and spermatocytes I in each patient’s sample. Then, we found the mean TL for one cell type for all of the patients, and calculated the deviation from the obtained mean value for each patient’s sample, expressing the obtained value as a number greater than 1 (that is, for values less than 1, we found the inverse of 1/x).

**Figure 4:**
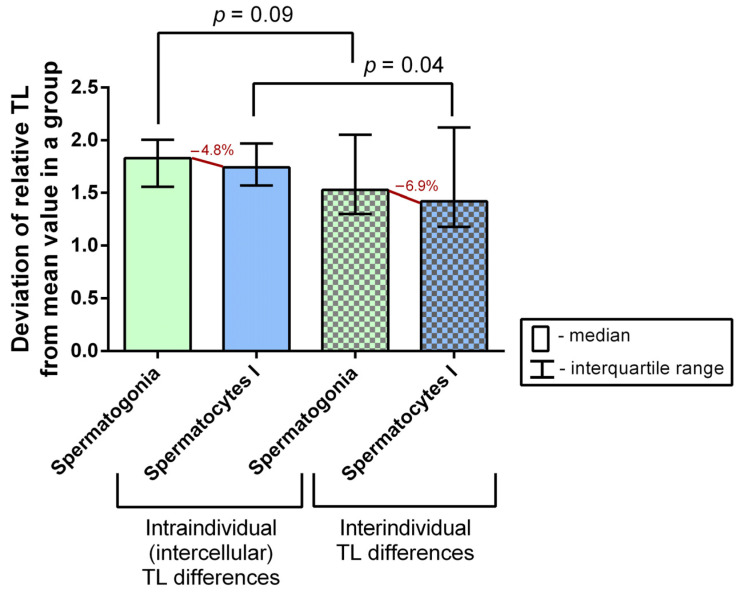
A comparison of intraindividual (intercellular) and interindividual variability of mean relative telomere lengths (TLs) in spermatogonia and spermatocytes I obtained from the testicular tissue samples of 30 azoospermic patients (Mann–Whitney U test).

The comparative analysis of intraindividual (intercellular) and interindividual relative TL variability did not show a significant difference in spermatogonia (Mann–Whitney U test, *p* = 0.09), but revealed a significant difference in spermatocytes I (Mann–Whitney U test, *p* = 0.04) (Figure 4). Therefore, spermatocytes I feature a significantly higher intraindividual (intercellular) than interindividual variability, which attests to the existence of multiple generations of spermatocytes I differing in TLs.

### 2.4. Analysis of Correlations between Azoospermic Patients’ Age and TL in Spermatogonia and Spermatocytes I

**Figure 5:**
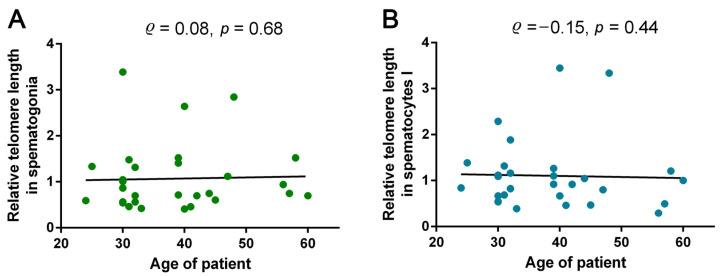
The correlations between relative telomere length (TL) in spermatogonia (**A**) and spermatocytes I (**B**) and azoospermic patients’ age (Spearman’s test, ρ = 0.08, *p* = 0.68 and ρ = −0.15, *p* = 0.44, respectively).

Considering the possible impact of azoospermic patients’ age on TL in spermatogonia and spermatocytes I, we investigated the correlations between these parameters. A correlation analysis did not show a significant dependence of TL in either spermatogonia (Spearman’s test, ρ = 0.08, *p* = 0.68) or spermatocytes I (Spearman’s test, ρ = −0.15, *p* = 0.44) on the patient’s age (Figure 5).

Therefore, with age, the length of telomeres in spermatogonia and spermatocytes I remains unchanged.

### 2.5. Analysis of Correlations between TL in Spermatogonia and Spermatocytes I and the Competence of Testicular-Sperm-Derived Embryos for In Vitro Preimplantation Development to the Morulae Stage

**Figure 6:**
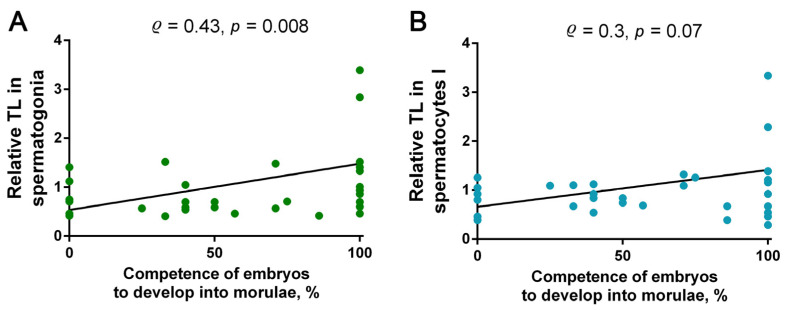
The correlations between the competence of embryos obtained through fertilization with testicular sperm from azoospermic patients’ testicular tissue samples for development to the morulae stage and the telomere length (TL) in spermatogonia (**A**: Spearman’s test, ρ = 0.43, *p* = 0.008) and spermatocytes I (**B**: Spearman’s test, ρ = 0.3, *p* = 0.07).

The use of azoospermic patients’ testicular sperm for ICSI yielded a total of 98 morulae-stage embryos for 23 couples (Table 1). To determine the possible correlation between the TL in spermatogonia and spermatocytes I and the embryo competence for on-time in vitro development to the morulae stage, i.e., from the second to the fourth day, we conducted a correlation analysis. Embryo competence for development was assessed as the ratio of the embryos that developed up to day four to the embryos that developed up to day two (Table 1). The correlation analysis showed a moderate positive correlation between the embryos’ competence for development and TL in spermatogonia (Spearman’s test, *p* = 0.008; ρ = 0.43) and a lack of a significant correlation with TL in spermatocytes I (Spearman’s test, *p* = 0.07, ρ = 0.3) (Figure 6). Therefore, the competence of testicular-sperm-derived embryos for in vitro development to the morulae stage depends, at least to some extent, on the TLs in spermatogonia.

### 2.6. Assessment of the Competence of Testicular-Sperm-Derived Embryos for In Vitro Development to the Blastocyst Stage Depending on TL in Spermatogonia and Spermatocytes I

**Figure 7:**
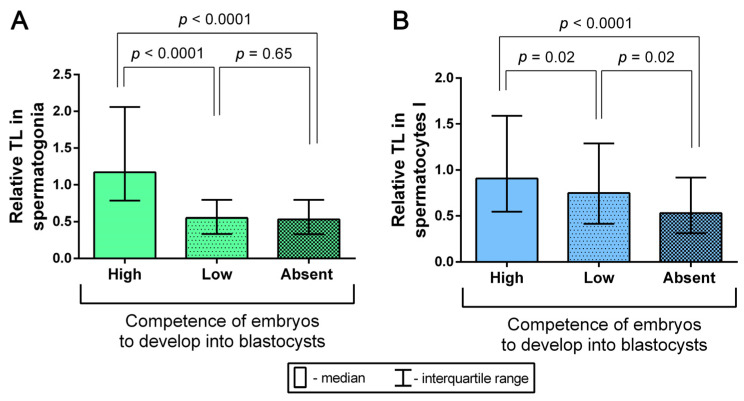
A comparison of median relative telomere length (TL) in spermatogonia (**A**) and spermatocytes I (**B**) from azoospermic patients’ biopsy samples across three groups, formed based on the competence of testicular-sperm-derived embryos from each couple for in vitro development to the blastocyst stage. The competence of the couple’s embryos for development was characterised as high if 50% or more two-cell embryos developed to the blastocyst stage (*n* = 7), low if less than 50% of the embryos reached that stage (*n* = 12) and absent if none of the embryos developed to the blastocyst stage (*n* = 6) (Mann–Whitney U test).

Having analyzed 37 embryological outcomes, we assessed the competence of testicular-sperm-derived embryos for development to the blastocyst stage for each of 25 married couples (Table 1). In five of the couples, testicular spermatozoa were not used for fertilization because the partners opted not to start the ICSI cycle. The competence of the couple’s embryos for development was assessed as high if 50% or more two-cell embryos reached the blastocyst stage in vitro, and as low if less than 50% of embryos developed to blastocysts in vitro. In cases when none of the couple’s embryos reached the blastocyst stage in vitro, the competence for development was treated as absent. A high embryo competence for development to the blastocyst stage was registered in seven cases; in twelve cases, a low competence was registered; and in six cases, none of the embryos reached the blastocyst stage (Table 1). The couples were grouped according to their embryos’ competence for development: high, low or absent. For each group with a high or low embryo competence for development to the blastocyst stage and for the group without blastocysts, we determined the median relative TLs in spermatogonia and spermatocytes I. A comparative analysis showed a significant increase in relative TL in spermatogonia in the high-competence group, compared to both the low-competence group (Mann–Whitney U test, *p* < 0.0001) and the group without blastocysts (Mann–Whitney U test, *p* < 0.0001) (Figure 7A). Similar differences between the examined groups were observed with regard to dependence on TL in spermatocytes I. Thus, the length of telomeres in spermatocytes I was significantly higher in the group with a high embryo competence for development to the blastocyst stage than in the low-competence group (Mann–Whitney U test, *p* = 0.02) and the group without blastocysts (Mann–Whitney U test, *p* < 0.0001) (Figure 7B). Therefore, we established that the competence of testicular-sperm-derived embryos for in vitro development to the blastocyst stage depends on the length of telomeres in spermatogonia and spermatocytes I. Consequently, an increase in TL in spermatogonia and spermatocytes I contributes to preimplantation embryo development to the blastocyst stage.

### 2.7. Analysis of the Relationship between TLs in Spermatogonia and Spermatocytes I and the Outcomes of Pregnancy and Birth after Testicular-Sperm-Derived Embryo Transfer

**Figure 8:**
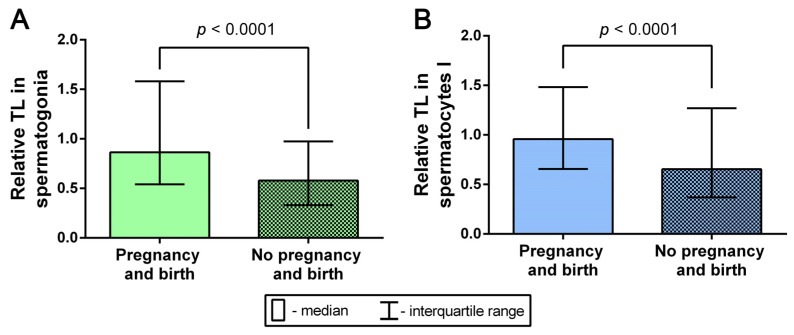
A comparative analysis of telomere lengths (TLs) in spermatogonia (**A**) (*n* = 99 and *n* = 204) and spermatocytes I (**B**) (*n* = 96 and *n* = 163) from the testicular biopsy samples of the patients whose testicular sperm yielded embryos that were transferred to the uterus with subsequent pregnancy and birth, and without subsequent pregnancy (Mann–Whitney U test).

According to the embryological outcome reports, in 22 of the cases, the testicular-sperm-derived embryos were transferred to the uterus. In thirteen cases, pregnancy did not occur; in nine cases, pregnancy did occur, resulting in childbirth in eight of the cases and in ectopic pregnancy in one case (Table 1). We conducted a comparative analysis of relative TLs in spermatogonia from the testicular biopsy samples of the eight patients whose testicular sperm was used for fertilization with subsequent transfer, pregnancy, and birth, and those of the thirteen patients whose testicular sperm was used for fertilization that did not result in pregnancy after embryo transfer. The comparison of TLs in spermatogonia showed a significant increase in the cases of successful pregnancies that ended in birth (Mann–Whitney U test, *p* < 0.0001) (Figure 8). The comparative analysis of TLs in spermatocytes I also showed a significant increase in telomere length in the cases of successful pregnancy and birth (Mann–Whitney U test, *p* < 0.0001) (Figure 8). Therefore, an increase in TL in both spermatogonia and spermatocytes I is associated with the occurrence of pregnancy and birth after the transfer of embryos obtained with the use of testicular sperm from azoospermic patients’ testicular tissue biopsy samples.

### 2.8. Analysis of the Female Partner’s Age Impact on the Occurrence of Birth after Testicular-Sperm-Derived Embryo Transfer to the Uterus

**Figure 9:**
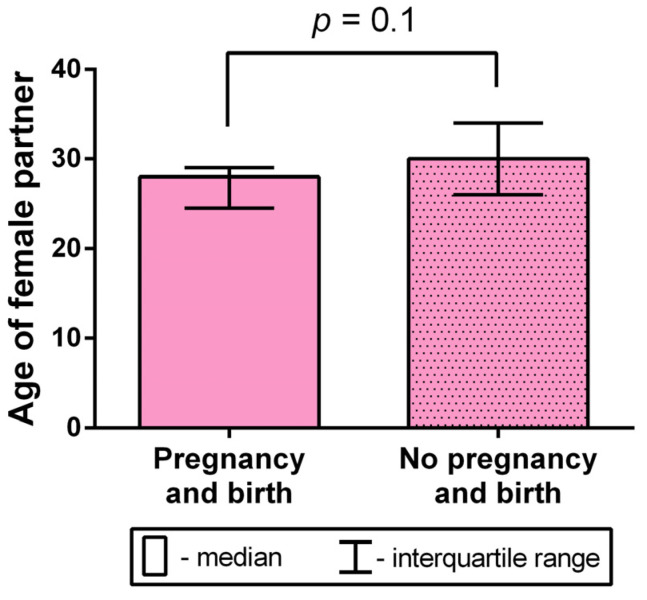
A comparative analysis of the female partner’s age between groups with and without occurrence of birth after the transfer of testicular-sperm-derived embryos to the uterus (Mann–Whitney U test).

Considering the possible impact of the female partner’s age on the occurrence of birth, we conducted a comparative mean age analysis in the group of women who became pregnant after the transfer of a testicular-sperm-derived embryo to the uterus (*n* = 6) and the group of women in which pregnancy did not occur (*n* = 21). The age of the female partners varied from 23 to 29 years old in the group with successful pregnancies (with a mean age of 27 ± 1 years old), and from 21 to 41 years old in the group without pregnancies (with a mean age of 30.29 ± 1.12 years old). In the pooled sample, the female partners’ age varied from 21 to 41 years old, with a mean age of 29.56 ± 0.93 years old. The comparison did not yield a significant difference (Mann–Whitney U test, *p* = 0.1), although the group without pregnancies featured a trend of older age (Figure 9).

Therefore, in the present study, the female partner’s age had no significant impact on the occurrence of birth after testicular-sperm-derived embryo transfer to the uterus.

## 3. Discussion

The length of telomeres in gametes is undoubtedly among the key factors determining the potential vitality of the resulting embryo. Telomeres in human oocytes have been shown to be the shortest [31,32,33] and telomeres in sperm have been shown to be the longest among all of the examined human cells [34,35,36]. As we have shown in the present study, TL increases by 24% in the course of spermatogenesis from spermatogonia to spermatocytes I. Most likely, this occurs through telomerase activity [37,38,39,40]. However, as the present study suggests, whereas TL in spermatogonia is characterized by low variability within a single sample, spermatocytes showed more pronounced intercellular variability. The latter may be the reason why a single organism is capable of forming germ cell generations with varying TLs. Admittedly, along with the varying efficiency of telomerase activity in spermatogonia, intercellular variability may be influenced by exogenous factors, which can impact any of the spermatogenesis stages. An excess of reactive oxygen species (ROS) is among the negative factors affecting TL [17,18,19,41,42,43]. For the reasons specified above, a single patient’s ejaculate may contain spermatozoa with considerably different TLs [40,44,45].

An intriguing fact that is nevertheless difficult to explain is the increase in TL in ejaculated spermatozoa with the man’s age [44,46,47,48]. To date, the specific mechanism responsible for this phenomenon is yet to be identified. In our study, we did not detect changes in TL in either spermatogonia or spermatocytes associated with an increase in the patient’s age. This could be the case because the present study examined the correlation between age and TL in spermatogenic cells whereas other studies showed an increase in TL with the man’s age examining ejaculated spermatozoa [44,46,47,48]. There is a possibility that age-dependent changes in TL occur during meiosis II or in subsequent spermatogenesis stages, which would require further research to investigate.

In the present study, we detected the influence of TL in spermatogenic cells on embryo competence for preimplantation development. The increase in TL in spermatogonia and spermatocytes I contributes to successful zygote development to the blastocyst. Therefore, TL in sperm-derived chromosomes is an important factor in determining embryo competence for preimplantation development. One of the key preimplantation development events is the pronuclear formation, in which TL plays an important part. Considering that telomeric regions in a spermatozoon are located at the nuclear periphery [49,50,51,52,53], they are the first to interact with the oocyte cytoplasm and respond to oocyte signals. Successful interaction is facilitated by the chromatin structure of telomeric regions, which is characterized by the presence of sperm telomere binding proteins (STBPs), a sperm-specific variant of H2B histones, and not protamine, which modulates the chromatin structures of other sperm DNA sequences [50,51,53,54]. Consequently, longer telomeres facilitate male pronuclear formation after fertilization, which is one of the key preimplantation development checkpoints. Furthermore, during the cleavage divisions, the length of telomeres in sperm-derived chromosomes also appears important in light of their involvement in the alternative lengthening of telomeres [55,56]. Most likely, telomeres in sperm-derived chromosomes serve as a matrix for oocyte-derived telomeres. Alternative telomere lengthening is necessary for the period when cell divisions are intensive but telomerase is not yet active—that is, before the blastocyst stage. TL also plays a significant part in correct chromosome disjunction during intensive mitotic activity in the preimplantation development stages [36,57,58,59]. There is ample evidence that this period is often fraught with chromosome disjunction errors, which result in the formation of mosaic aneuploid embryos with low potential vitality [60,61,62,63,64]. The latter is one of the main causes of poor ART outcomes [61,64].

The formation of a morphologically normal blastocyst and its successful implantation in the uterus results in a pregnancy. We have demonstrated that the length of telomeres in spermatogenic cells has a significant impact on this event. Thus, we found that the TL in both spermatogonia and spermatocytes I was significantly higher in cases when the transfer of testicular-sperm-derived embryos into the uterus resulted in birth than in cases when it did not. The obtained result may be used for the development of a prognostic test based on the assessment of TL in spermatogenic cells for predicting ART cycle outcomes. The use of spermatogonia and spermatocytes for the assessment of telomere lengths has an incontestable advantage of not involving procedures that are traumatic for the embryo or the sex cells used for fertilization. Once the required number of spermatozoa is obtained, testicular biopsy fragments are not used in the later stages of the procedure and can be studied to determine TL, and possibly other parameters relevant to predicting the ART cycle outcome. Therefore, determining TLs in spermatogenic cells is likely to have prognostic value for ART cycles that use the testicular spermatozoa of azoospermic patients for fertilization.

Advantages and limitations of the methodology used in the present study should be noted. The present study assessed TLs with the use of the well-established Q-FISH technique, applied in a variety of studies [65,66,67,68,69], including our recent research on human uterine leiomyoma cells [70], chorionic cytotrophoblast cells [30], PHA-stimulated lymphocytes [71] and tripronucleate zygotes [23]. Q-FISH has several incontestable advantages. First, the technique detects the location and size of target sequences on individual chromosomes and cells, providing the unique opportunity of working with individual cells of the same type. Second, it allows for a series of hybridization tours with different probes on the same sample. To increase the accuracy of Q-FISH and mitigate the impact of factors such as the efficiency of the hybridization process per se and the varying degrees of chromatin compaction, we normalized the measurement of fluorescent signal intensity. To that end, we obtained the ratios of the average fluorescence intensity of probes to telomere regions to the average reference probe fluorescence intensity. It should also be acknowledged that the analysis of the relative TL assessed with Q-FISH is widely recognized as sufficiently accurate; in combination with the TRF (terminal restriction fragment length) analysis (a method based on the digestion of genomic DNA, except for telomeric repeats, by restriction enzymes and further determining the length of telomeric repeats by Southern blot), it is considered to be the gold standard of TL assessment, with a strong positive correlation of results [66]. Therefore, using the Q-FISH technique is a reliable approach to assessing the TL of individual chromosomes in spermatogenic cells.

Our analysis was performed exclusively on the ‘direct’ (without cell culturing) preparations of mitotic and meiotic chromosome spreads, i.e., on dividing in vivo cells. On the one hand, this approach ensured that the results of TL analysis closely reflected the situation in vivo. On the other hand, the number of obtained chromosome spreads on ‘direct’ preparations was never high, which, at least in part, restricts the sample size. Manual evaluation of telomeric and subtelomeric fluorescence on mitotic and meiotic chromosomes enabled us to exclude the background noise and perform accurate analysis. However, the possible number of measurements is also restricted in the case of manual analysis. Finally, our study was performed on the samples of human testicular tissue that are primarily obtained for clinical purposes of ART and are not often readily available for experimental procedures, which limited the number of patients enrolled in the study.

To summarize, our study suggests that the length of telomeres in spermatogenic cells can be considered a prognostic criterion in the assessment of the competence of testicular-sperm-derived embryos for pre- and post-implantation development. Considering the availability of spermatogonia and spermatocytes obtained from TESE biopsy samples for research, the development of a laboratory test based on telomere length analysis appears practical after thorough analysis of the revealed relationships on a larger sample. The test may be used for the prognosis of the clinical outcomes of ART cycles using testicular spermatozoa. Assessing the risks of failure at various ART stages based on the results of the proposed test and understanding their causes will contribute to more efficient planning of ART cycles.

## 4. Materials and Methods

### 4.1. Collection of Human Testicular Biopsy Samples

The testicular samples were obtained at the D.O. Ott Research Institute of Obstetrics, Gynaecology and Reproductology (Saint Petersburg, Russia) via TESE carried out on 30 azoospermic patients. After the testicular tissue extraction, spermatozoa were collected and cryopreserved for future use in ART treatment. The remaining testicular tissue was transferred to the culture medium (G-MOPS, Vitrolife, Gothenburg, Sweden) and stored at room temperature for no longer than 1 h until treatments for chromosome preparation were applied.

### 4.2. Chromosome Preparation

For cytogenetic preparation of mitotic and meiotic chromosome spreads from testicular tissue samples, a ‘direct’ technique (i.e., without culturing) was applied according to the protocol for ‘direct’ preparations from tissue fragments repeatedly used in our earlier studies [72,73,74]. Briefly, a testicular tissue sample was separated into small fragments and placed for two hours at room temperature into 0.9% sodium citrate solution containing colchicine (Merck, Rahway, NJ, USA) at a final concentration of 2.5 μg/mL. Then, 2.5 mL of the solution was removed and a fixative (three parts methanol, one part acetic acid) was added drop by drop. Ten minutes later, all solution was removed and 5 mL of a cold fixative was added for at least 2 h at −20 °C. To obtain the chromosome spreads, a fragment of testicular tissue from the fixative was placed on blotting paper and then transferred to a drop of 60% acetic acid solution on a glass slide. After maceration in a drop of 60% acetic acid, the remaining fragments were removed, and the drop with the cell suspension was evenly spread over the glass slide and fixed with several drops of fixative. The preparations were aged at +55 °C for 12–24 h before their further use in the experimental procedures.

### 4.3. Fluorescence In Situ Hybridization (FISH)

Fluorescence in situ hybridization (FISH) with telomeric probes (K532611-8, DAKO, Glostrup, Denmark) was carried out to detect telomeric regions on the chromosome preparation slides. All of the procedures were performed according to the manufacturer’s recommendations, with minor modifications, as described in our earlier study [23].

After photoimaging, the 19p subtelomeric region (the reference region for TL measurements in our study) was detected on the same metaphase preparations through FISH with a Vysis TelVysion SpectrumGreen 19p DNA probe (Abbott Laboratories, Chicago, IL, USA).

### 4.4. Image Acquisition and the Evaluation of Telomeric and 19p Subtelomeric FISH Probe Signal Intensity

The fluorescence images of chromosomes after FISH were acquired using the Leica Application Suite V.3.8.0 software on a Leica DM 2500 microscope with a Leica DFC345 FX camera (Leica Microsystems, Wetzlar, Germany). All fluorescence images of chromosomes with telomeric DNA probes were acquired using the following settings: exposure time, 7.0 s; gain, ×3.2; and gamma, 1.75. All fluorescence images of chromosomes with 19p reference DNA probes were acquired with the following settings: exposure time, 7.0 s; gain, ×1; and gamma, 2.01. The FISH signal intensities of the telomeric and 19p subtelomeric probes on the images were evaluated using the Image J V.1.52n software, which enabled the signal intensity to be measured in the selected area of the image. Each signal area was manually selected with the Freehand Selection tool of Image J V.1.52n. Each pixel in the selected area of the image was automatically assigned a value from 0 to 255, depending on its grayscale value in 8-bit mode. The average fluorescence intensity of the selected area was calculated automatically by Image J V.1.52n by summing the values assigned to all pixels and dividing the resulting sum by the number of pixels.

### 4.5. Statistical Analysis

Statistical analysis was carried out using the GraphPad Prism software, Version 6.01 (GraphPad Software, San Diego, CA, USA). The correlation coefficients were estimated by the nonparametric Spearman’s test. Differences between nonparametric variables were estimated using the Mann–Whitney U test.

## Figures and Tables

**Table 1:** The participants’ demographic details, relative telomere lengths (TLs) in spermatogenic cells and embryological outcomes.

# Couple	Azoospermic Patient’s Age	Number of Sperm in TESE Sample Field of View	Relative TL		Female Partner’s Age	# ICSI Protocol	Embryological Outcomes								
			In Spermatogonia	In Spermatocytes I			2p Zygotes	4-Cell Embryos on Day 2	8-Cell Embryos on Day 3	Morulae on Day 4	Blastocysts on Day 5	Embryo Competence for Development into Blastocyst	Embryo Transfer to the Uterus	Pregnancy Occurrence	Birth Occurrence
1	30	5–10	1.009	0.544	24	I	3	3	3	3	3	High	Yes	No	No
2	30	3–5	3.387	2.287	27	I	4	4	4	4	1	High	Yes	No	No
					29	II	6	6	6	6	6		Yes	No	No
3	58	>10	1.520	1.207	25	I	2	1	1	1	1	High	Yes	Yes	Yes
4	39	3–5	1.407	0.923	28	I	0	0	0	0	0	High	No	No	No
					29	II	6	6	6	6	3		Yes	Yes	Yes
5	25	5–7	1.334	1.390	24	I	2	2	2	2	2	High	Yes	No	No
					24	II	2	2	2	2	0		* Yes	No	No
6	30	4–5	0.863	0.669	28	I	5	4	4	4	4	High	Yes	Yes	Yes
7	31	2–3	1.482	1.318	23	I	7	7	5	5	5	High	Yes	Yes	Yes
8	24	1–2	0.592	0.842	25	I	7	2	1	1	1	Low	Yes	No	No
					26	II	10	5	3	2	2		Yes	Yes	Yes
9	30	2–3	0.565	1.090	33	I	6	4	1	1	0	Low	No	No	No
					35	II	7	7	5	5	4		Yes	No	No
10	30	2	1.045	1.119	28	I	7	5	5	2	2	Low	Yes	Yes	Yes
11	31	3–5	0.463	0.688	26	I	10	7	7	4	2	Low	Yes	No	No
12	33	5–10	0.423	0.390	31	I	7	7	6	6	5	Low	Yes	Yes, ectopic	No
					29	II	3	0	0	0	0		No	No	No
13	42	8	0.698	0.917	29	I	5	5	2	2	2	Low	Yes	No	No
14	39	8–10	0.714	1.264	32	I	4	4	3	3	3	Low	Yes	No	No
					33	II	1	0	0	0	0		* Yes	Yes	Yes
15	40	3–5	0.408	0.667	30	I	9	6	2	2	0	Low	* Yes	No	No
					30	II	8	7	7	6	1		Yes	No	No
16	39	2–3	1.520	1.101	36	I	7	3	3	1	1	Low	Yes	No	No
17	45	>10	0.603	0.469	33	I	3	3	3	3	1	Low	Yes	No	No
					37	II	0	0	0	0	0		No	No	No
18	30	10	0.542	0.539	35	I	5	5	2	2	2	Low	No	No	No
19	48	5–7	2.844	3.339	29	I	4	4	4	4	0	Low	No	No	No
					29	II	4	1	1	1	0		No	No	No
					29	III	4	1	1	1	1		Yes	Yes	Yes
20	41	2–3	0.459	0.462	21	I	1	0	0	0	0	Absent	*** Yes	No	No
					37	II	2	0	0	0	0		* Yes	No	No
					N/A	III	5	2	2	2	0		* Yes	No	No
21	44	4	0.748	1.046	38	I	2	1	0	0	0	Absent	No	No	No
22	32	2–3	0.698	1.161	30	I	12	10	10	10	0	Absent	* Yes	No	No
23	47	10	1.118	0.800	41	I	1	0	0	0	0	Absent	** Yes	No	No
24	56	1	0.941	0.291	32	I	7	6	6	6	0	Absent	No	No	No
25	60	3–4	0.700	0.741	37	I	2	2	1	1	0	Absent	* Yes	No	No
26	57	>10	0.748	0.496	-	-	-	-	-	-	-	-	-	-	-
27	42	5–7	0.681	0.919	-	-	-	-	-	-	-	-	-	-	-
28	32	0	0.561	0.824	-	-	-	-	-	-	-	-	-	-	-
29	32	1	1.315	1.884	-	-	-	-	-	-	-	-	-	-	-
30	40	1–2	2.643	3.447	-	-	-	-	-	-	-	-	-	-	-

*, Morula on Day 5; **, 8-cell embryo on Day 5; ***, 4-cell embryo on Day 5; N/A, data not available.

## Data Availability

The data are available by contacting the corresponding author.
